# Mindfulness training enhances face working memory: evidence from the drift-diffusion model

**DOI:** 10.1038/s41539-025-00389-0

**Published:** 2025-12-09

**Authors:** Hui Kou, Wei Luo, Xiaodong Li, Jia Wu, Qianguo Xiao, Taiyong Bi

**Affiliations:** 1 Research Center of Humanities and Medicine, Zunyi Medical University, Zunyi, China; 2 The Institute of Ethnology and Anthropology, Chinese Academy of Social Sciences, Beijing, China

**Keywords:** Human behaviour, Psychology

## Abstract

The effect of mindfulness training on working memory is unclear. The current study sought to confirm the impact of mindfulness training on working memory for facial stimuli and to reveal the cognitive mechanisms underlying this effect by using drift-diffusion modeling (DDM). Using a delayed match-to-sample task with facial stimuli, we measured memory performance across five emotional categories (happy, sad, fearful, angry, neutral). Sixty participants received five-week emotion-targeted mindfulness training versus 60 waitlist controls. Assessments pre-training, post-training, and at one-month follow-up revealed significantly improved memory accuracy for all emotions except fear, with the effects persisting for one month. More importantly, drift-diffusion modeling showed increased drift rates across emotional categories post-training. Furthermore, accuracy improvements strongly correlated with drift rate enhancements within each emotion category. These findings demonstrate that mindfulness training induces lasting improvements in both accuracy and processing efficiency of visual working memory, independent of facial emotions, clarifying its cognitive mechanisms.

## Introduction

Mindfulness is a state characterized by bringing intentional awareness to present internal and external experiences with curiosity, openness, acceptance, and without judgment, free from entanglement in rumination about past experiences and anxieties about the future^1,2^. From this perspective, mindfulness fundamentally involves two core components: (1) intentionally directing attention to present experiences, and (2) adopting an accepting attitude toward these experiences. The human mind relies on a set of cognitive processes, such as attention and working memory, to guide its moment-to-moment experiences. However, when these processes become abnormal, it can lead to difficulties in coping with life’s challenges. Mindfulness training serves as a form of mental exercise that improves these fundamental cognitive processes^3^. Mindfulness training can enhance meta-awareness by consciously monitoring thoughts, emotions, and behaviors, which contribute to modifying maladaptively automatic cognitive patterns and disrupting maladaptively automatic reaction patterns to stimuli^4–6^. A large number of studies revealed that mindfulness training can alter a variety of cognitive processes, including various forms of perception^7–9^, attention^10–14^, and memory^15–17^.

Working memory plays a core role in cognitive processing, providing temporary storage and manipulation of the information necessary for cognitive tasks^18^. The efficacy of working memory can significantly influence one’s ability to process information, thereby impacting overall cognitive performance and daily functioning. Baddeley^19^ proposed that working memory comprises the central executive system, two subsidiary slave systems—the phonological loop and the visuospatial sketchpad, and the episodic buffer^19^. The central executive system, an attentional control system, is capable of retrieving information from storage in the form of conscious awareness. Holas and Jankowski propose a cognitive model of mindfulness that emphasizes the cognitive processes shaping a state of mindfulness, with the executive functions of attention and working memory components identified as their determinants^20^. However, previous findings regarding the effects of mindfulness training on working memory are mixed. Although a large number of studies have revealed that mindfulness training improves working memory^21–26^, some other studies did not find such an effect^27–30^. Therefore, it is necessary to examine the effect of mindfulness training on working memory processing, and related influencing factors. One of the possible factors that can influence working memory is emotion. The valence, arousal, and motivational dimensions of emotional content can all influence working memory performance^31^. It was found that emotional expressions facilitated working memory^32^. Evidence showed that the performance of working memory was lower for neutral and happy faces than for negative faces^33,34^. However, other studies have revealed that identity recognition accuracy is higher for happy faces than for angry faces in a 2-back face identity matching task^35^, and working memory performance is faster for happy than for neutral and sad faces^36^. Nevertheless, there is a study showing that task accuracy remains unaffected by the emotional content of the stimuli^37^. In a modified Recency-probes paradigm, both the valence and arousal of emotional stimuli facilitate interference resolution in working memory^38^. Furthermore, mindfulness training may be able to reduce emotional reactivity by fostering non-judgmental awareness. Previous studies have shown that mindfulness reduces emotional reactivity to emotional stimuli^39–42^. This dampening of emotional interference frees attentional resources, potentially enhancing working memory for both emotional and neutral content. To test whether emotion moderates mindfulness-related working memory improvements, we examined the impact of mindfulness training on working memory across five facial emotion categories (happy, sad, fearful, angry, neutral) using drift-diffusion modeling to disentangle cognitive efficiency from emotional bias in the present study. Given mindfulness training’s established role in reducing emotional reactivity, we further hypothesized that training-induced improvements would be emotion-independent. Specifically, mindfulness training would enhance the accuracy and processing efficiency of working memory across emotional categories.

Working memory can be assessed using the delayed match-to-sample (DMTS) task^43–46^, in which one or more stimuli are presented as a sample (encoding), and after a delay period (maintenance), the effectiveness of maintaining the sample information is assessed by presenting a test stimulus and requiring participants to indicate whether the test stimulus matches the sample (retrieval)^45^. Previous studies have indicated that the DMTS task exhibits stable test-retest scores^47^ and shares common underlying networks of working memory^48^. Our task followed a classic three-phase structure: (1) Encoding phase: Two sample faces (emotional or neutral) were presented for 1000 ms. (2) Delay phase: A 2000 ms blank interval to prevent sensory persistence. (3) Retrieval phase: A single test face was presented centrally, and participants indicated via key press whether it matched one of the sample faces.

**Fig. 1:**
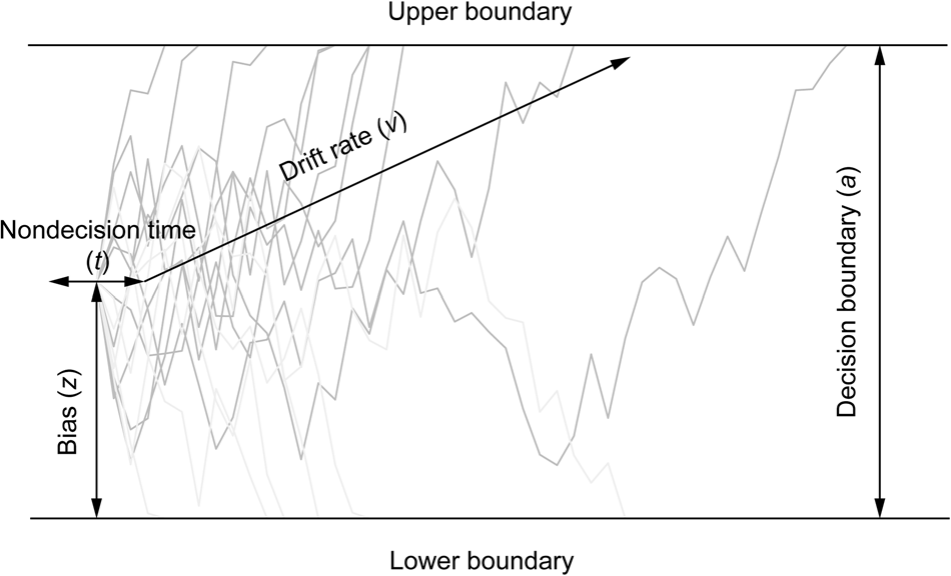
Demonstration for the drift-diffusion model. Drift rate (*v*) represents the average evidence accumulation speed. Decision boundary (*a*) represents the difference between boundaries. Bias (*z*) represents the starting point of evidence accumulation. Nondecision time (*t*) represents the time not used for the process of decision-making.

Traditionally, cognitive processing was assessed by response accuracy and time. However, these behavioral indicators include components beyond cognition. The drift-diffusion model (DDM), a perceptual decision-making model, can separate different components from observed behavioral variables^49,50^. The DDM is widely applied to fit the evidence-accumulation process of various tasks^51–56^. It hypothesizes that individuals stochastically accumulate evidence from a starting point until reaching a decision boundary, triggering a choice. Fig. 1 characterizes the model and illustrates key parameters. Four model parameters are fitted. Drift rate (*v*) represents the average evidence accumulation speed. A higher *v* indicates a lower task difficulty or higher ability. Decision boundary (*a*) represents the difference between boundaries. Higher *a* increases accuracy but slows responses. Bias (*z*) represents the starting point of evidence accumulation. Nondecision time (*t*) represents the time not used for the process of decision-making (e.g., motor execution). Studies show training can alter DDM parameters, like increasing drift rates^57^ or decreasing boundary separation^58^. However, no comprehensive studies have specifically examined which DDM parameters mindfulness training affects. Given mindfulness’s characteristics, it is interesting to explore if it influences *v* (cognitive processing) or *t* (non-reacting). While mindfulness benefits working memory and cognitive control, the mechanisms are unclear. The DDM provides a robust framework for decomposing decision processes into distinct components. Results from the present study could significantly enhance understanding of how mindfulness impacts the working memory mechanism.

## Results

All the results for each test phase are presented in the supplementary materials (Table S1, Table S2, Table S3).

### Mindfulness

Immediately after training, the training group showed a significantly larger improvement in dispositional mindfulness than the control group (*t*(118) = 2.26, *p* = .026, Cohen’s d = 0.41, 95% CI [0.42, 0.41]). This effect persisted one month after training (*t*(118) = 2.39, *p* = 0.018, Cohen’s d = 0.44, 95% CI [0.50, 5.27]). The results indicate that the mindfulness training enhanced the level of mindfulness effectively.

### Accuracy and RT

We first calculated the improvements in accuracies, RTs for the correct responses, and d’ immediately after training (T2-T1) and one month after training (T3-T1).

Regarding the T2-T1 improvements in accuracies, independent samples t test showed significantly larger improvements for the training group than the control group for the happy (*t*(118) = 2.79, *p* = 0.031, Cohen’s d = 0.51, 95% CI [0.01, 0.08], Bonferroni corrected), sad (*t*(118) = 4.05, *p* < 0.001, Cohen’s d = 0.74, 95% CI [0.03, 0.08], Bonferroni corrected), angry (*t*(118) = 3.13, *p* = 0.011, Cohen’s d = 0.57, 95% CI [0.02, 0.08], Bonferroni corrected), and neutral (*t*(118) = 2.89, *p* = 0.023, Cohen’s d = 0.53, 95% CI [0.01, 0.08], Bonferroni corrected) faces. However, the difference was nonsignificant for the fearful face (*t*(118) = 1.33, *p* = 0.187, Cohen’s d = 0.24, 95% CI [–0.01, 0.06], uncorrected).

**Fig. 2:**
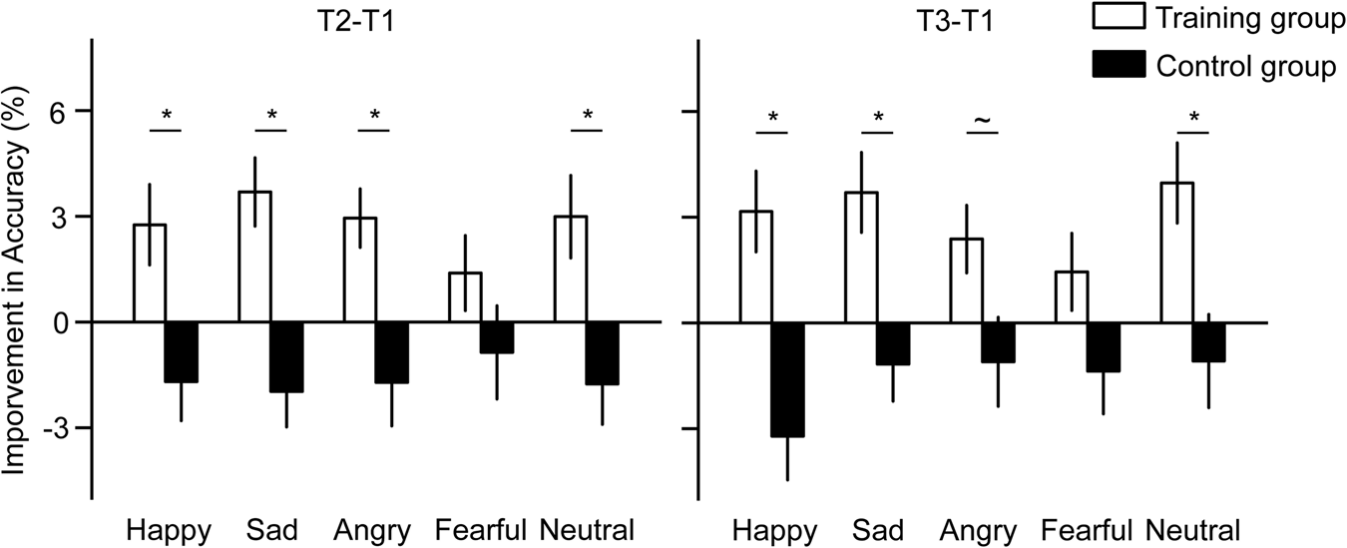
The improvements in the accuracy in the delayed match-to-sample task. Improvements were calculated by subtracting the baseline (pre-training) accuracies from the post-training accuracies. Error bars denote one standard error of the mean. *: *p* < 0.05, Bonferroni corrected; ~: *p* < 0.05, uncorrected.

Regarding the T3-T1 improvements in accuracies, independent samples t test showed significantly larger improvements for the training group than the control group for the happy (*t*(118) = 3.77, *p* = 0.001, Cohen’s d = 0.69, 95% CI [0.03, 0.10], Bonferroni corrected), sad (*t*(118) = 3.16, *p* = 0.010, Cohen’s d = 0.58, 95% CI [0.02, .08], Bonferroni corrected), and neutral (*t*(118) = 2.89, *p* = 0.023, Cohen’s d = 0.53, 95% CI [0.02, 0.09], Bonferroni corrected) faces. The difference was marginally significant for the angry face (*t*(118) = 2.20, *p* = 0.030, Cohen’s d = 0.40, 95% CI [0.003, 0.07], uncorrected) and nonsignificant for the fearful face (*t*(118) = 1.73, *p* = 0.087, Cohen’s d = 0.32, 95% CI [–0.004, 0.06], uncorrected). The results for the improvements in accuracies are presented in Fig. 2.

Regarding the RTs, no significant difference was found between groups for T2-T1 improvements (all *t* < 2.2, *p* > 0.05, Bonferroni corrected), as well as T3-T1 improvements (all *t* < 1.1, *p* > 0.05, Bonferroni corrected).

Besides, we also calculated the d’ for the DMTS task according to the Signal Detection Theory. The descriptive statistics and between-group difference for d’ were reported in Table S1 and Table S3. In summary, the training group showed significantly larger improvements in sad and angry faces at T2 (both *t* > 2.8, *p* < 0.05, Bonferroni corrected), and in sad and happy faces at T3 (both *t* > 3.1, *p* < 0.05, Bonferroni corrected).

### Modeling parameters

Four parameters, including drift rate (*v*), decision boundary (*a*), initial bias (*z*), and nondecision time (*t*), were extracted. Improvements in these parameters were calculated immediately after training (T2-T1) and one month after training (T3-T1).

**Fig. 3:**
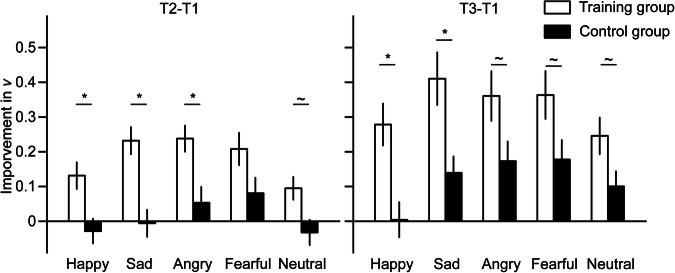
The improvements of the drift rate (*v*) in the delayed match-to-sample task. Improvements were calculated by subtracting the baseline (pre-training) *v* from the post-training *v*. Error bars denote one standard error of the mean. *: *p* < 0.05, Bonferroni corrected; ~: *p* < 0.05, uncorrected.

Drift rate is the core parameter in DDM, which reflects the cognitive processing of evidence accumulation. The results of improvements in *v* are presented in Fig. 3.

Regarding the T2-T1 improvements in *v*, independent samples ttest showed significantly larger improvements for the training group than the control group for the happy (*t*(118) = 3.05, *p* = 0.014, Cohen’s d = 0.56, 95% CI [0.06, 0.26], Bonferroni corrected), sad (*t*(118) = 4.32, *p* < 0.001, Cohen’s d = 0.79, 95% CI [0.13, 0.35], Bonferroni corrected), and angry (*t*(118) = 3.12, *p* = 0.011, Cohen’s d = 0.57, 95% CI [0.07, 0.30], Bonferroni corrected) faces. The difference was marginally significant for the neutral face (*t*(118) = 2.60, *p* = 0.010, Cohen’s d = 0.48, 95% CI [0.03, 0.22], uncorrected) and nonsignificant for the fearful face (*t*(118) = 1.97, *p* = 0.051, Cohen’s d = 0.36, 95% CI [–0.0003, 0.25], uncorrected).

Regarding the T3-T1 improvements, independent samples t test showed significantly larger improvements for the training group than the control group for the happy (*t*(118) = 3.48, *p* = 0.004, Cohen’s d = 0.64, 95% CI [0.12, 0.43], Bonferroni corrected) and sad (*t*(118) = 3.04, *p* = 0.015, Cohen’s d = 0.55, 95% CI [0.09, 0.45], Bonferroni corrected) faces. The difference was marginally significant for angry (*t*(118) = 2.06, *p* = 0.042, Cohen’s d = 0.38, 95% CI [0.01, 0.37], uncorrected), fearful (*t*(118) = 2.09, *p* = 0.039, Cohen’s d = 0.38, 95% CI [0.01, 0.36], uncorrected), and neutral (*t*(118) = 2.13, *p* = 0.035, Cohen’s d = 0.39, 95% CI [0.01, 0.28], uncorrected) faces.

Similarly, we compared the group difference in the improvements in other parameters. At T2, the training group showed larger improvements in *t* for fearful (*t*(118) = 3.10, *p* = 0.012, Cohen’s d = 0.57, 95% CI [0.02, 0.08], Bonferroni corrected) and neutral (*t*(118) = 3.71, *p* = 0.002, Cohen’s d = 0.68, 95% CI [0.03, 0.11], Bonferroni corrected) faces. At T3, the training group only showed a larger improvement in *t* for the neutral face (*t*(118) = 3.11, *p* = 0.012, Cohen’s d = 0.57, 95% CI [0.02, 0.11], Bonferroni corrected). All other differences in other parameters were nonsignificant (all *t* < 1.8, *p* > 0.05, Bonferroni corrected).

### Correlations between accuracy and ν

As mindfulness training enhanced both the memory accuracy and drift rate, to investigate the associations between the training-induced improvements in these two indicators, we calculated the correlation coefficients between them among individuals in the training group. Results are presented in Table 1. In summary, the accuracy and *v* had a close relationship within each face category. However, the relationship between categories was weak.

**Table 1:** Correlations between improvements in accuracy and *v*

T2-T1						T3-T1				
	Happy_*v*	Sad_*v*	Angry_*v*	Fearful_*v*	Neutral_*v*	Happy_*v*	Sad_*v*	Angry_*v*	Fearful_*v*	Neutral_*v*
Happy_Accuracy	0.88***	0.22	0.06	0.01	0.26*	0.70***	0.27*	0.20	0.05	0.04
Sad_Accuracy	0.14	0.81***	0.10	0.08	0.24	0.24	0.53***	0.03	0.03	–0.04
Angry_Accuracy	0.23	0.04	0.73***	0.15	0.04	0.16	0.15	0.57***	0.19	–0.02
Fearful_Accuracy	0.04	0.03	0.21	0.83***	–0.04	–0.11	0.10	–0.03	0.61***	–0.07
Neutral_Accuracy	0.18	0.15	–0.15	–0.10	0.88***	–0.13	–0.17	–0.17	–0.11	0.53***

^*^p < 0.05, ***p < 0.001.

## Discussion

Our study demonstrates that mindfulness training significantly enhances working memory accuracy for faces. Meanwhile, DDM analysis revealed accelerated evidence accumulation speed after training, indicating improvements in cognitive processing. Notably, these cognitive improvements exhibit sustained effects over time, underscoring their potential as an intervention for enhancing cognitive performance across diverse populations. Furthermore, we identified a robust positive correlation between working memory accuracy gains and accelerated evidence accumulation, demonstrating that training improves both the precision of face memory and the efficiency of underlying cognitive processes. This converging evidence from accuracy and drift rate results advances our knowledge of the effect of mindfulness training on the working memory process.

In the present study, we found a beneficial impact of mindfulness training on the enhancement of working memory. Previous studies have also indicated that individuals who undergo mindfulness training exhibit a significant increase in their capacity to retain and manipulate information in working memory, such as working memory capacity^24–26,59–61^, accuracy^62,63^, and updating^60,64–66^. The Mindful Coping Model suggests that mindfulness brings about a “decentered” metacognitive state that enhances cognitive flexibility, thereby breaking free from automatic reactive mode, and initiating an adaptive response^67,68^. The Mindfulness-to-Meaning Theory further posits that mindfulness can neutralize initial cognitive appraisals for stressors in the face of adversity by disrupting the automatic activation of schemata to free one’s focus of attention from rumination on stressors, and foster more flexible and adaptive cognitive processes^69^. Empirical evidence exhibits that mindful decentering protects the ability to clear irrelevant information from working memory under negative emotional conditions^70^, and higher working memory capacity predicts greater self-distancing (e.g., decentering)^71^. Therefore, mindfulness training is an effective tool to enhance individuals’ working memory.

However, the traditional analysis of behavioral performance could not distinguish the changes in cognitive processes and other processes such as response bias or motion execution. The present study utilized the DDM analysis to demonstrate that the changes in *v* contributed a lot to the training effect on memory accuracy. Meanwhile, mindfulness training does not significantly affect other DDM parameters such as *t*, *z*, and *a*. This suggests that mindfulness training, which targets attentional control and metacognitive awareness, primarily influences the efficiency of cognitive processing rather than other processes during working memory tasks. The *a* reflects the amount of evidence required before making a response, with higher values indicating a more cautious response strategy. Evidence showed that DDM boundary effects may be more commonly linked to task instructions (e.g., speed-accuracy tradeoffs) than to mindfulness training^58^. Stable *a* in the present study suggests mindfulness training did not alter response caution. The *z* represents a tendency to favor one response (e.g., match vs. non-match) before evidence accumulation begins. Starting-point changes may be primarily produced by changes in prior probability and potential payoff^72,73^. Stable *z* in the present study suggests that mindfulness training did not induce response biases. The *t* captures processes unrelated to evidence accumulation, such as stimulus encoding, motor preparation, and response execution. In the present study, the training group exhibited larger *t* increases in neutral faces at T2 and T3, indicating prolonged processing of these preparation stages. However, these effects disappeared in emotional faces, suggesting that *t* is not central to the cognitive mechanism underlying mindfulness training. This interpretation aligns with prior studies showing that mindfulness can not alter nondecision time^74,75^. However, the influence of facial expressions on nondecision time remains controversial. Some studies revealed emotion-specific effects on the nondecision time^76^, while others did not find these effects^77^. A potential explanation for the lack of consistent *t* effects in our study lies in the prioritized processing of emotional stimuli, which may weaken mindfulness-related modulation of nondecision time. Emotional stimuli are inherently prioritized in cognitive and motivational systems^78^, and even when emotional expression is irrelevant to the identity-matching task, emotional faces trigger automatic emotion regulation, diverting cognitive resources from motor preparation and response execution. Neurobiologically, this is supported by evidence that emotional faces enhance functional integration between the amygdala and premotor cortices compared to neutral faces^79^. These findings suggest that nondecision time is a context-dependent measure sensitive to emotional salience, rather than a core marker of mindfulness training effects. These separated parameter outcomes highlight DDM’s capacity to distinguish various components within complex tasks, a critical advantage over conventional behavioral indicators.

Furthermore, we found a robust correlation between accuracy and drift rate for faces sharing identical emotional expressions, suggesting these metrics are rooted in shared underlying mechanisms, confirming the DDM’s remarkable efficacy in cognitive task modeling. The drift rate in the DDM reflects the efficiency of evidence accumulation. The strong convergence of accuracy and drift rate may indicate that mindfulness training simultaneously enhances the precision and efficiency of information processing, both of which share the core cognitive resource. A recent study revealed that drift rate fully mediated the effect of trait anxiety on accuracy in a novel paradigm that combines N-back and Stroop tasks, also revealing the close association between the two factors^80^. Previous studies have demonstrated that mindfulness training may enhance processing speed^81,82^ and simultaneously improve decision-making accuracy^83–85^. Consistently, studies concerning the neural correlates of mindfulness training also revealed enhancements in indicators related to the cognitive processing of working memory. For example, electrophysiological evidence suggested that mindfulness training increased P3 amplitude and theta power at electrode site Pz during a 3-back task^60^. Neuroimaging evidence suggested that mindfulness training enhanced working memory performance, while increasing brain activation in the bilateral inferior parietal lobule, right posterior insula, and right precuneus^66^. However, it is worth noting that there are other studies showing a beneficial effect of mindfulness training on other processes. For example, a previous study found that meditators showed a modest overall increase in their decision threshold, which may reflect an ability to wait longer and collect more information before responding during the attention network task^75^. In conclusion, in the present delayed match-to-sample task, we revealed that the beneficial effect of mindfulness training on working memory may be mainly attributable to changes in processing efficiency.

Another important finding of the present study is that the training effect on working memory may not be restricted to faces with specific expressions. We note that the training effect was least evident for fearful faces in the accuracy results. However, the drift rate results revealed a moderate training effect on the fearful faces. A possible explanation for the mere improvement in memory accuracy could be the ceiling effect due to a relatively high baseline accuracy for the fearful face at T1. However, through DDM analysis, we could still reveal a moderate training effect for the fearful face. Although only moderate effects were found for some expressions, our results may reveal a nonspecific cognitive enhancement by mindfulness training, suggesting that the training appears to drive a broad-based improvement in the foundational mechanisms of cognitive processing linked to working memory. Although emotional content was found to affect working memory^38,86–88^, mindfulness may reduce the interference of emotional content on working memory. Mindfulness may potentially free attentional resources typically consumed by affective interference through reducing emotional reactivity, which allows more efficient allocation of working memory resources, and thereby enhances overall working memory performance across all emotional faces. A lot of studies have revealed that mindfulness training reduces emotional reactivity. For example, mindfulness training was associated with attenuated subjective impact of emotional stimuli as well as amygdala deactivation during emotion processing^39^, and reduced amygdala response to emotional face distractors^89^ as well as to emotional pictures^42^. However, it’s worth noting that emotional congruency between the sample and test faces in our DMTS task may modulate the current findings. When the test face matches a sample face, it shares both emotional valence and facial identity with the encoded stimuli. This overlap raises the possibility that performance could reflect perceptual familiarity rather than active working memory maintenance. But from a different perspective, this design is beneficial for controlling interference with memory retrieval of facial identity, which is caused by changes in facial muscles resulting from emotional differences between sample faces and the test face. Future studies should adopt modified designs to isolate memory processing, such as using neutral faces as probes^90,91^. In conclusion, changes in general working memory related to mindfulness are likely multifaceted, and mindfulness may erect a firewall that shields cognitive processing resources from intrusions by emotional stimuli.

Finally, it is worth noting that there were very weak correlations between enhancements in the working memory of different emotional faces. Several reasons may account for the null findings. First, it may indicate that independent processing mechanisms for different emotions. However, it is still debated whether the processing of basic emotions is independent. Some neuroimaging studies demonstrated distinct neural mechanisms across emotional expressions. For example, there were different activation patterns in the amygdala and frontal cortex when processing fear versus happiness expressions^92–94^ as well as fear versus anger^95^; the left amygdala, right orbitofrontal cortex, and temporal cortices were involved in the processing of the angry or disgusted expressions, while the right angular gyrus was involved in the processing of happy expressions^96^. However, there is also evidence that does not support independent processing of different emotions. A perceptual learning study revealed confusion between disgust and anger, as well as the confusion between fear and surprise^97^. Other studies have also found confusion among the six basic emotions. For example, confusion between fear and surprise has been observed in populations from different cultural backgrounds^98–102^. Considering the muscular movements involved in the process of generating facial expressions, there is a significant overlap in the movements between expressions of disgust and anger as well as fear and surprise^103^. Besides, we should note that the weak relationships among the training effects across different face categories do not definitively demonstrate independent emotion-processing mechanisms, as task-specific factors (e.g., perceptual demands of facial feature matching) may also contribute to limited cross-emotion covariance. Future studies combining neuroimaging or electrophysiology with behavioral measures are needed to directly test whether emotional expressions engage distinct neural substrates during mindfulness-enhanced working memory.

There are several limitations in the present study. First, mindfulness training was viewed as a unitary construct without dissociating its core components, such as focused attention, emphasizing engagement with the selected object and disengagement from task-irrelevant distractions and open monitoring, emphasizing moment-by-moment attention to whatever arises in one’s conscious experience, without focusing or elaborating on the content of any particular object^23^. It may preclude definitive conclusions about whether specific subprocesses (e.g., attentional control vs. emotion regulation) differentially mediate working memory enhancement. Previous studies revealed that open monitoring, rather than focused attention, selectively elicits a more cautious and intentional response style, characterized by increased accuracy, slower reaction times, and diminished P3 amplitude during the Eriksen flanker task^104^. Direct evidence revealed that, compared to mindfulness-based socioemotional training and mindfulness-based sociocognitive skills, only mindfulness-based attention training enhanced working memory performance^105^. Future studies need to focus on the effect of discrete facets of mindfulness training.

Second, the present study only focuses on working memory accuracy in the delayed match-to-sample paradigm, which overlooks complementary dimensions of working memory, including capacity, updating, interference susceptibility, etc. Furthermore, the load of working memory was set at one level (i.e., two emotional faces) in the present study. Future research should incorporate multi-task batteries (e.g., the n-back task for updating, the delayed-match-to-sample task for maintenance, and the flanker task for interference control) to examine mindfulness training’s impacts on the multifacet of working memory.

Third, the exclusive use of facial expressions as emotional stimuli restricts generalizability to broader affective processing domains. Mindfulness may have different impacts on working memory for non-facial emotional stimuli. A previous study revealed that the mindfulness group remembered fewer negative words compared to the control group, but there was no difference in the recall of positive words between the two groups^106^. Systematic comparisons across stimulus modalities in future work could clarify whether the benefits of mindfulness training on working memory reflect domain-specific adaptations or generalized affective processing enhancements.

Fourth, training duration may modulate the effect of mindfulness training. Previous studies demonstrated that working memory performance was affected by mindfulness training with enough training dose^25,105,107^; and brief mindfulness breathing exercises were not sufficient to enhance working memory capacity^30^. In the present study, we utilized long-duration mindfulness training. Further studies are required to investigate the impact of mindfulness training with different training doses on working memory, and the effect of emotions.

Fifth, although we ruled out participants with self-reported history of neurological or psychiatric illness, we did not formally evaluate participants’ subclinical symptoms of anxiety or depression using standardized scales. Subclinical emotional symptoms could potentially influence working memory performance^108,109^ or mindfulness training responsiveness^110^. Future studies will include validated measures of anxiety and depression to control for baseline emotional states and to stratify participants by symptom severity, enabling subgroup analyses of mindfulness effects in emotionally vulnerable populations.

The last limitation is that the probe stimulus shared emotional valence with sample faces, potentially conflating perceptual familiarity with working memory maintenance. Future studies should use neutral probes to isolate memory processes.

## Methods

### Participants

Sample size was calculated a priori using GPower 3.1 (effect size = 0.50, power = 0.80, α = 0.05). A total sample size of 102 participants was required to detect a significant between-group difference in an independent t test. One hundred and twenty undergraduates were recruited from a medical university by the recruitment advertisement. They were randomly divided into the training group (50 females, age 20.27 ± 1.30) and the control (untrained) group (40 females, age 20.00 ± 0.88). All of the participants were right-handed and had normal or corrected-to-normal vision and normal color vision, and none of them had a history of neurological or psychiatric illness. All participants got paid for participation after completing all tasks.

### Measurements

The Five Facet Mindfulness Questionnaire (FFMQ) was used to measure the mindfulness of participants^111^. It contains 39 items and encompasses five subscales, including observing (e.g., “When I take a shower or bath, I stay alert to the sensations of water on my body”), describing (e.g., “I can easily put my beliefs, opinions, and expectations into words”), acting with awareness (e.g., “When I do things, my mind wanders off and I’m easily distracted”), non-judging (e.g., “I tell myself I shouldn’t be feeling the way I’m feeling”) and non-reacting (e.g., “When I have distressing thoughts or images, I just notice them and let them go”). All of the items were rated on a 5-point Likert scale from 1 (very rarely true) to 5 (always true). The Cronbach’s α of the FFMQ was 0.70 in a Chinese non-clinical sample^112^.

### Materials

Digital color pictures depicting emotional faces were adopted from the Chinese Facial Affective Picture System (CFAPS)^113^. 24 angry faces, 24 sad faces, 24 happy faces, 24 fearful faces, and 24 neutral emotional faces were selected from the system. All pictures had a uniform size (185*200 pixels; visual angle: 5.80°×6.27° at a viewing distance of 60 cm) and were matched for brightness and contrast. The valence for angry, sad, happy, fearful, and neutral faces is 2.70, 3.01, 6.38, 2.84, and 4.31 respectively, while the arousal was 6.18, 5.64, 5.57, 6.49, and 3.85 respectively. ANOVA revealed that the emotional valence was significantly different among face categories (*F(4, 115)* = *206.33, p* < 0.001). Post hoc analysis showed that the emotional valence of the happy face was higher than other faces (all *ps* < *.05*, Bonferroni corrected). In addition, the valence of angry, sad, and fearful faces was not significantly different from each other (all *ps* > *0.05*, Bonferroni corrected), which was significantly lower than the neutral face (all *ps* < *0.05*, Bonferroni corrected). Regarding arousal, ANOVA showed a significant difference among categories (*F(4, 115)* = *19.51, p* < *0.001*). Post hoc analysis showed that the arousal was significantly lower for the neutral face than other faces (all *ps* < *0.05*, Bonferroni corrected), while the difference among the four emotional faces was nonsignificant (all *ps* > *0.05*, Bonferroni corrected).

### Apparatus

The visual stimuli were presented on a SAMSUNG 19-in LCD screen, with a spatial resolution of 1280 × 800 and a refresh rate of 60 Hz^114^. The subjects viewed the stimuli from a distance of 60 cm. The presentation of stimuli was controlled by E-Prime 2.0 software (https://www.pstnet.com). Data analysis was conducted via SPSS 16.0 (https://www.ibm.com/analytics/SPSS-statistics-software).

### Procedure

The protocol of the training program included four phases. The first phase is the baseline test (T1). The second phase is the training phase, in which the training group received a five-week mindfulness training while the control group received two mindfulness lectures. The third phase is the post-training test (T2). The fourth phase is the test one month after the training (T3). The measurements in the three test phases were totally the same for both groups.

**Fig. 4:**
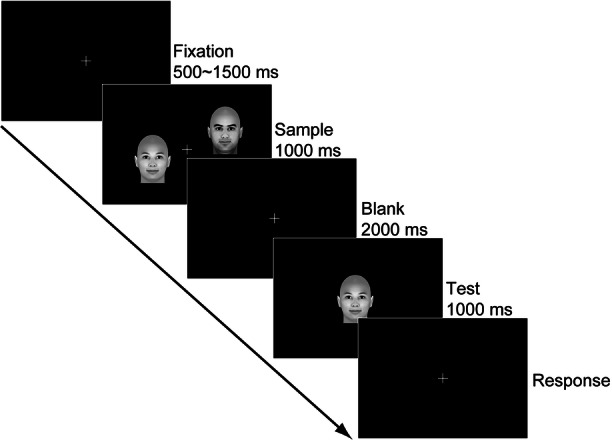
An example of the experimental procedure of the delayed match-to-sample task. Two faces (sample) appeared at two of the four quadrants for 1000 ms. After a delayed period of 2000 ms, a test face was presented at the center of the screen for 1000 ms. Participants were asked to judge if the test face matched one of the sample faces.

At T1, all participants first completed the scale and were then tested with a delayed match-to-sample (DMTS) task in a quiet room. At the beginning of each trial, a white fixation cross was presented in the center of the black screen for a random period of 500 –1500 ms. Then, two faces appeared at two of the four quadrants for 1000 ms. After the disappearance of the sample faces, a blank interval of 2000 ms was presented. Then, a test face was presented at the center of the screen for 1000 ms. Then, the response screen is presented until participants respond. Participants were asked to press one key (F) if the test face matched one of the sample faces and another key (J) if they didn’t match as quickly and accurately as possible. There was no explicit time limit for keypress responses. Keypress responses were permitted both during and after the test face presentation. After the response, a blank screen was presented for 500 ms, and then the next trial began. Each block consisted of 48 trials, in half of which the test face matched the sample face. The emotion of faces in one block was the same across trials. Therefore, each participant completed five blocks, with each block consisting of happy, sad, angry, fearful, and neutral faces respectively. Trial types and the locations of the sample faces were randomly presented in a block, and the block types were counterbalanced among participants. An example of the procedure is demonstrated in Fig. 4.

After the baseline measurement, participants were randomly divided into the training group that received mindfulness training or the control group that attended two lectures about mindfulness. Participants in the training group had systematically participated in the five-week course of mindfulness-based stress reduction (MBSR) under the guidance of a therapist who had three years of experience in leading MBSR courses and long-term meditation practice. The mindfulness training program was designed based on the MBSR^2^ and mindfulness-based cognitive therapy (MBCT)^115^. To specifically enhance emotional perception and emotional regulation, the contents of each weekly training focused on the topic of emotion. In each week, participants received a two-hour training course. The contents of the weekly training are shown in Table 2. The control group attended two lectures on mindfulness, which were designed to concentrate on general principles of mindfulness (e.g., definitions, benefits, and everyday applications) and did not include experiential practices. Each lecture lasted 60 min and was conducted by the same instructor leading the MBSR courses. After the training, participants were asked to complete daily mindfulness practice at home and report their feelings.

**Table 2:** The contents of weekly mindfulness training

Sessions	Topics	Course contents
Preparation	Course introduction	Introduction of the content, form, duration, and rules of the mindfulness intervention course, including respect, confidentiality, non-judgment, etc.
First week	The first experience with mindfulness	Understanding what mindfulness is by eating raisins with mindfulness and mindfulness body scan.
Second week	Mindfulness and emotional recognition	Recognizing emotions (cognitive evaluation, physiological arousal, subjective feelings, behavioral manifestations, and responses) - recognition, mindful awareness, and experience.
Third week	Mindfulness and emotional cognition	Understanding the relationships between emotional experience and cognition and reactions, learning the attitude of allowing and letting go.
Fourth week	Mindfulness and emotional response	Identifying the experience and body responses to emotion, learning the attitude of non-reactivity.
Fifth week	Mindfulness and emotion regulation	Flexible mindfulness response, emotional process, and course summary

Immediately after the training (T2), participants were asked to complete the post-training measurement that was the same as the baseline measurement, i.e., the FFMQ scale and the DMTS task. The same test was repeated one month after the training (T3).

After all the measurements were completed, the control group also received mindfulness training if they volunteered to participate.

### Statistical analysis

In this study, we measured the level of mindfulness with the FFMQ scale. Therefore, the scores of the scale were first analyzed.

Regarding the DMTS task, behavioral responses were recorded. Mean accuracy and mean reaction time (RT) for the correct responses were calculated for each condition. Then, the discrimination index (d’) for the DMTS task was calculated according to the Signal Detection Theory. Hit rate (H) and False alarm rate (FA) were first calculated, and d’ was calculated as d’ = Z(H) - Z(FA).

Furthermore, a drift-diffusion model (DDM) was constructed based on the responses and RTs in each condition. We utilized the dockerHDDM^116^ platform to perform the Bayesian hierarchical drift-diffusion modeling. Four basic parameters in the DDM were extracted for each condition and each subject: drift rate (*v*), decision boundary (*a*), initial bias (*z*), and nondecision time (*t*). In the model fitting, the sample number was set to 10000 samples with 5000 burn-ins to accurately estimate parameters and ensure convergence in hierarchical modeling^116^. The upper bound of the model is the correct response while the lower bound is the incorrect response.

Before statistical comparisons, we calculated the improvement for each indicator as we focused on the effect of mindfulness training. The scale score, behavioral performance, and modeling parameters before training (at T1) were treated as baselines and were subtracted from the indicators after training (T2 or T3). Specifically, the improvement of an indicator was calculated as the post-training indicator minus the pre-training indicator (T2-T1 or T3-T1). Such manipulation could largely reduce the complexity of our analysis and make our results clear to present. Furthermore, following the group difference analysis, we aimed to investigate the relationship between the training-induced changes in the behavioral performance and modeling parameters, which requires computing the improvements in these indicators. Therefore, the following analyses were all based on the improvements in the indicators.

Afterward, we compared the improvements between the two groups with independent-sample t-tests. The Bonferroni adjustment was applied to address the multiple comparisons issue. Furthermore, to investigate the relationship between the improvements in accuracy and drift rate (*v*), Pearson correlation coefficients were calculated between the two indicators among individuals in the training group.

### Ethics approval

All procedures performed in this study involving human participants were in accordance with the ethical standards of the Ethical Committee of Human Research at Zunyi Medical University (reference number: [2024]1-046) and with the 1964 Helsinki Declaration and its later amendments or comparable ethical standards. Informed consent was obtained from all participants before participation.

## Supplementary information

Supplementary Information

## Data Availability

The data supporting this work can be found at https://osf.io/c5x2q/.

## Code availability

Custom code or scripts in the generation or analysis of the datasets can be shared upon reasonable request by the corresponding author. Sample size was calculated a priori using GPower 3.1 (https://www.psychologie.hhu.de/arbeitsgruppen/allgemeine-psychologie-und-arbeitspsychologie/gpower). The presentation of stimuli was controlled by E-Prime 2.0 software (https://www.pstnet.com). Data analysis was conducted via SPSS 16.0 (https://www.ibm.com/analytics/SPSS-statistics-software). The Bayesian hierarchical drift-diffusion modeling was performed via dockerHDDM^116^.
