# Supplementary material for: Mindfulness training enhances face working memory: evidence from the drift-diffusion model
Source: NPJ Sci Learn. 2025 Dec 9;11:7. doi: 10.1038/s41539-025-00389-0 (PMC12804977; doi:10.1038/s41539-025-00389-0)
Supplement: Supplementary file 1 — Supplementary Information [file 41539_2025_389_MOESM1_ESM.pdf]

Supplementary materials

Table S1 FFMQ scores (M±SD)

	T1	T2	T3
Training group	86.55±9.94	89.63±10.68	89.50±10.18
Control group	85.23±8.75	84.90±8.47	85.90±7.94

FFMQ: The Five Facet Mindfulness Questionnaire

Table S2 Behavioral performance (M±SD)

Face category	T1		T2		T3	
	Training group	Control group	Training group	Control group	Training group	Control group
Accuracy (%)						
Sad	84.10±6.83	80.75±7.52	87.79±6.28	78.78±9.83	87.81±6.34	79.57±9.15
Angry	85.56±6.62	82.45±8.09	88.51±6.04	80.74±9.08	87.94±6.91	81.35±9.28
Happy	79.48±7.38	76.67±7.93	82.24±7.32	74.98±9.38	82.65±8.79	73.46±10.03
Fearful	86.69±7.05	81.98±8.61	88.08±5.83	81.11±8.12	88.14±6.65	80.61±9.76
Neutral	74.91±7.69	72.67±8.27	77.90±8.45	70.91±9.69	78.88±8.14	71.58±9.99
RT (ms)						
Sad	889.50±223.88	896.00±194.24	849.83±235.12	844.05±207.77	741.04±98.73	792.24±182.43
Angry	869.38±179.27	911.56±257.50	842.16±190.54	821.64±195.37	748.86±118.65	796.70±164.91
Happy	913.61±229.66	916.98±233.56	891.09±259.87	846.07±207.55	787.40±121.77	847.01±280.88

Fearful	878.06±232.91	916.51±246.11	849.34±230.49	827.49±195.43	745.04±109.86	797.68±167.83
Neutral	929.62±273.92	968.92±289.37	919.46±258.73	858.55±218.81	805.86±132.64	807.44±203.57
d'						
Sad	2.19±0.59	1.94±0.60	2.55±0.61	1.82±0.78	2.56±0.62	1.88±0.70
Angry	2.33±0.62	2.07±0.67	2.60±0.63	1.96±0.72	2.59±0.68	2.05±0.69
Happy	1.81±0.61	1.62±0.57	2.04±0.62	1.54±0.68	2.11±0.69	1.44±0.66
Fearful	2.46±0.65	2.06±0.69	2.57±0.61	1.97±0.61	2.57±0.67	2.03±0.71
Neutral	1.50±0.56	1.33±0.56	1.71±0.63	1.24±0.66	1.77±0.60	1.30±0.67

RT: Reaction time

Table S3 Modeling parameters (M±SD)

Face category	T1		T2		T3	
	Training group	Control group	Training group	Control group	Training group	Control group
v						
Angry	1.44±0.23	1.29±0.30	1.67±0.27	1.34±0.37	1.80±0.57	1.46±0.46
Fearful	1.45±0.27	1.25±0.28	1.65±0.28	1.33±0.33	1.81±0.54	1.43±0.49
Happy	1.04±0.24	0.95±0.24	1.17±0.28	0.93±0.32	1.32±0.50	0.96±0.45
Sad	1.33±0.25	1.19±0.26	1.56±0.31	1.19±0.34	1.74±0.56	1.33±0.43
Neutral	0.83±0.24	0.74±0.24	0.92±0.27	0.71±0.30	1.07±0.46	0.84±0.36
a						
Angry	1.40±0.18	1.45±0.26	1.36±0.23	1.34±0.22	1.28±0.17	1.29±0.16
Fearful	1.44±0.21	1.44±0.24	1.37±0.25	1.35±0.23	1.28±0.16	1.28±0.18

Happy	1.38±0.21	1.38±0.23	1.34±0.29	1.30±0.23	1.25±0.16	1.25±0.20
Sad	1.39±0.21	1.40±0.22	1.37±0.22	1.35±0.25	1.25±0.16	1.26±0.17
Neutral	1.38±0.24	1.43±0.26	1.34±0.25	1.33±0.24	1.22±0.17	1.24±0.19
t						
Angry	0.49±0.10	0.49±0.09	0.49±0.11	0.46±0.08	0.46±0.06	0.46±0.08
Fearful	0.48±0.08	0.50±0.10	0.49±0.12	0.46±0.09	0.46±0.06	0.46±0.07
Happy	0.49±0.07	0.50±0.12	0.50±0.07	0.48±0.10	0.48±0.05	0.46±0.08
Sad	0.49±0.08	0.49±0.08	0.48±0.11	0.47±0.08	0.46±0.06	0.46±0.08
Neutral	0.48±0.07	0.50±0.13	0.51±0.11	0.46±0.09	0.50±0.07	0.46±0.11
z						
Angry	0.50±0.001	0.50±0.001	0.50±0.001	0.50±0.001	0.49±0.001	0.49±0.002
Fearful	0.51±0.001	0.51±0.001	0.50±0.001	0.50±0.001	0.49±0.001	0.49±0.001
Happy	0.51±0.002	0.51±0.002	0.52±0.001	0.52±0.001	0.51±0.001	0.51±0.002
Sad	0.50±0.001	0.50±0.001	0.50±0.001	0.50±0.001	0.50±0.001	0.50±0.002
Neutral	0.52±0.002	0.52±0.001	0.53±0.001	0.53±0.001	0.51±0.001	0.51±0.002

Table S4 The between-group differences in the improvements in d'

Face category	T2-T1					T3-T1				
	t	*p*	Cohen's d	95%CI		*t*	*p*	Cohen's d	95%CI	
				Lower	Upper				Lower	Upper
Sad	3.88	<0.001	0.71	0.24	0.73	3.15	0.002	0.58	0.16	0.70

Angry	2.83	0.006	0.52	0.12	0.66	2.15	0.034	0.39	0.02	0.55
Happy	2.33	0.022	0.43	0.05	0.57	3.62	<0.001	0.66	0.21	0.73
Fearful	1.29	0.200	0.24	-0.10	0.49	0.94	0.350	0.17	-0.15	0.41
Neutral	2.52	0.013	0.46	0.06	0.54	2.25	0.026	0.41	0.03	0.55
